# Factors Affecting Unmet Need Contraception in Patients with Autoimmune Disease in Dr. Cipto Mangunkusumo Hospital

**DOI:** 10.12688/f1000research.167793.1

**Published:** 2025-08-22

**Authors:** Lisa Novianti, Adeprita Pratiwi Herlinawati, Lathifah Nabilahsari, Aisyah Retno Puspawardani, Intan Indah Permatasari, Nafi'atul Ummah, Suzy Maria, Achmad Kemal Harzif

**Affiliations:** 1 Department of Obstetrics and Gynecology, Faculty of Medicine, University of Indonesia, Dr.Cipto Mangunkusumo Hospital, Jakarta, 10330, Indonesia; 2 Human Reproduction, Infertility, and Family Planning Cluster, Indonesia Reproductive Medicine Research and Training Center, Faculty of Medicine, Universitas Indonesia, Jakarta, 10330, Indonesia; 3 Department of Internal Medicine, Faculty of Medicine, Universitas Indonesia, Dr.Cipto Mangunkusumo Hospital, Jakarta, 10330, Indonesia; 4 Reproductive Immunoendocrinology Division, Department of Obstetrics and Gynecology,, Faculty of Medicine Universitas Indonesia, Dr. Cipto Mangunkusumo General Hospital, Jakarta, 10330, Indonesia

**Keywords:** Autoimmune, Unmet Need, Contraception, Family Planning

## Abstract

**Aim & Background:**

Women with autoimmune diseases usually require contraception. Various factors influence women with autoimmune diseases who do not use contraceptive methods. However, there has been no research on the unmet need for contraception in patients with autoimmune diseases. Therefore, this study aimed to determine the relationship between sociodemographic factors and unmet need for contraception in patients with autoimmune diseases.

**Methods:**

This was a cross-sectional study of female patients aged 15-49 years who had autoimmune diseases at the Internal Medicine Clinic of Dr. Cipto Mangunkusumo Hospital. The subjects were then asked to complete the informed consent form and questionnaire. Statistical analyses were performed using the chi-square test for bivariate analyses.

**Results:**

We included 92 subjects with a mean age of 35.6±6.26 years old, with an age range was 25-48 years old. Twenty (21.7%) subjects had an unmet need for contraception, while 72 (69.4%) used contraception or were planning for pregnancy. We found that education level (p=0.017) and Internet usage frequency (OR 3.42; 95%CI 1.02-11.45; p=0.037) were significantly associated with unmet need contraception in females with autoimmune disease.

**Conclusion:**

This study found that education level and Internet usage frequency were significantly associated with unmet need contraception in females with autoimmune disease.

**Clinical significance:**

The findings emphasize that education and Internet access are vital for meeting the contraceptive needs of women with autoimmune diseases.

## Introduction

Contraception is an act of preventing conception using tools, chemicals, drugs, and even surgical procedures. ^1^ Unmet need for contraception refers to women who want to delay childbirth for at least two years or want to stop giving birth, but are not using any contraceptive method. ^2^ Globally, the prevalence of contraceptive use is increasing, but unmet need for contraception is still a problem. In 2019, 190 million married women reported an unmet need for contraception. The prevalence of unmet need contraception in Africa was 22% and that in Oceania was 15%, which is higher than that in other regions. ^3^ In Indonesia, family planning services have a major impact on maternal morbidity and mortality. Increasing the prevalence of contraceptive use and decreasing the prevalence of the unmet need for contraception in family planning services will significantly reduce maternal morbidity and mortality during pregnancy and childbirth. ^4^

Autoimmune disease is a condition in which a person’s immune system fails to recognize their body tissue, considers it a foreign object, and attacks it. Autoimmune diseases cause inflammation in parts of the body where they are attacked. Autoimmune diseases affect more women than men, with a ratio of 2:1. ^5^ Contraceptive and are classified based on their duration of action, reversibility, and mechanism of action as hormonal contraceptives and non-hormonal contraceptives. Autoimmune diseases can respond to hormonal stimulation (especially steroid sex hormones), and autoimmune diseases can be affected by pregnancy and the use of hormonal contraceptives, such as pills, patches, injections, implants, or intrauterine progestin-releasing systems. ^5^

Women with autoimmune diseases usually do not use contraceptives. A survey conducted in Finland found that contraceptive use was lower in patients with systemic lupus erythematosus (SLE) compared to healthy subjects of the same age. ^6^ When using contraception, the most recommended methods are barrier methods and natural methods. ^7^ A 2011 study of 206 women with SLE in the United States found that as many as 42% were at risk of experiencing an unwanted pregnancy, and almost two-thirds of them had never received an explanation of the contraceptive methods that could be used. ^8^

Various factors influence women with autoimmune diseases who do not use contraceptive methods. Additionally, research on the unmet need for contraception in the general population has been widely conducted. However, no research has been conducted on the unmet need for contraception in patients with autoimmune diseases. Therefore, this study aimed to determine the relationship between sociodemographic factors and unmet need for contraception in patients with autoimmune diseases.

## Methods

This was a cross-sectional study that used a consecutive sampling method with inclusion criteria specified for female patients, aged 15-49 years, who had autoimmune diseases. This study was conducted at the Internal Medicine Clinic of Dr. Cipto Mangunkusumo Hospital. Written informed consent was obtained from all participants prior to the commencement of the study. The subjects were asked to fill out an informed consent form and questionnaire. The participants were assured of their anonymity throughout the study to encourage honest responses. Participants were excluded if they were unwilling to participate in the study or if the questionnaire was incomplete. The minimum sample size in this study was determined using the unpaired categorical analysis formula; thus, the minimum sample size was 92 participants.

The instrument used in this study was a questionnaire. The questionnaire contained questions on informed consent, name, age, religion, education level, occupation, address, husband’s education level, husband’s occupation, number of live births, miscarriage, mobile phone ownership, Internet access, frequency of Internet use, exposure to information related to family planning, contraceptive use, and pregnancy plans. To minimize potential bias, different personnel were assigned to handle the distribution and collection of the questionnaires, and a separate team was responsible for data handling and analysis.

The data obtained from the questionnaire answers are presented in a table form. A univariate analysis was performed to determine the frequency and percentage of each variable. Bivariate analysis was performed using the Chi-square test to examine the relationship between demographic factors and unmet need for contraception in women with autoimmune diseases at Dr. Cipto Mangunkusumo Hospital.

## Results

We included 120 participants, but only 92 had complete data and were used for the analyses. The mean age was 35.6 ± 6.26 years, with a range being 25-48 years. Twenty (21.7%) subjects had an unmet need for contraception, while 72 (69.4%) were using contraception or planning for pregnancy. Most of the participants were Muslim (89.1%), bachelor’s (47.8%), housewives (55.4%), and lived in Jakarta (43.4%). All subjects had a handphone and access to the internet. Meanwhile, most husbands were bachelors (34.7%) and employees (65.2%). The subjects’ characteristics are shown in Table 1.

**Table 1.** Subjects characteristics.

Variables	Total n (%)	Unmet need contraception n (%)	Family planning n (%)
Total subjects	92 (100)	20 (21.7)	72 (69.4)
**Religion**			
Islam	82 (89.1)	19 (23.2)	63 (76.8)
Christian	9 (9.7)	1 (11.1)	8 (88.9)
Buddha	1 (0.2)	0 (0)	1 (100)
**Subjects’ Education Level**			
Elementary school	3 (3.2)	3 (100)	0 (0)
Middle school	9 (9.7)	2 (22.2)	7 (77.8)
High school	25 (27.7)	5 (20)	20 (80)
Diploma	8 (8.6)	3 (37.5)	5 (62.5)
Bachelor	44 (47.8)	7 (15.9)	37 (84.1)
Postgraduates	3 (3.2)	0 (0)	3 (100)
**Husbands’ Education Level**			
Elementary school	5 (5.4)	2 (40)	3 (60)
Middle school	3 (3.2)	1 (33.3)	2 (66.7)
High school	30 (32.6)	9 (30)	21 (70)
Diploma	12 (13)	4 (33.3)	8 (66.7)
Bachelor	32 (34.7)	3 (9.4)	29 (90.6)
Postgraduates	9 (9.7)	1 (11.1)	8 (88.9)
**Subjects’ Occupation**			
Housewife	51 (55.4)	16 (31.4)	35 (68.6)
Employee	26 (28.2)	2 (7.7)	24 (92.3)
Teacher	9 (9.7)	1 (11.1)	8 (88.9)
Others	6 (6.5)	1 (16.7)	5 (83.3)
**Husbands’ Occupation**			
Employee	60 (65.2)	13 (21.7)	47 (78.3)
Enterpreneur	17 (18.5)	5 (29.4)	12 (70.6)
Teacher	5 (5.4)	1 (20)	4 (80)
Others	10 (10.9)	1 (10)	9 (90)
**Address**			
Jakarta	40 (43.4)	10 (25)	30 (75)
BoDeTaBek	35 (38.1)	8 (22.9)	27 (77.1)
Others	17 (18.5)	2 (11.8)	15 (88.2)
**Mobile phone ownership**			
Yes	100 (100)	20 (21.7)	72 (78.3)
No	0 (0)	0 (0)	0 (0)
**Access to the internet**			
Yes	100 (100)	20 (21.7)	72 (78.3)
No	0 (0)	0 (0)	0 (0)
**Internet usage frequency**			
Rarely	14 (15.5)	6 (42.9)	8 (57.1)
Min. 1 times a week	78 (84.5)	14 (17.9)	64 (82.1)
**Exposure to family planning information**			
Never	38 (41.3)	11 (28.9)	27 (71.1)
Rarely	44 (47.8)	9 (20.5)	35 (79.5)
Min. 1 times a week	10 (10.9)	0 (0)	10 (100)

Data are presented by n (%).

From bivariate analysis, we found that subjects’ education level (p = 0.017) and Internet usage frequency (OR 3.42; 95%CI 1.02-11.45; p = 0.037) were significantly associated with unmet need contraception in females with autoimmune diseases. Other variables were not significantly associated with an unmet need for contraception in females with autoimmune disease. The results of bivariate analysis are presented in Table 2.

**Table 2.** Factors affecting unmet need contraception in autoimmune patients.

Variables	Unmet need contraception n (%)	Family planning n (%)	p value
**Religion**			
Islam	19 (23.2)	63 (76.8)	0.61
Christian	1 (11.1)	8 (88.9)	
Buddha	0 (0)	1 (100)	
**Subjects’ Education Level**			
Elementary school	3 (100)	0 (0)	*0.017
Middle school	2 (22.2)	7 (77.8)	
High school	5 (20)	20 (80)	
Diploma	3 (37.5)	5 (62.5)	
Bachelor	7 (15.9)	37 (84.1)	
Postgraduates	0 (0)	3 (100)	
**Husbands’ Education Level**			
Elementary school	2 (40)	3 (60)	0.237
Middle school	1 (33.3)	2 (66.7)	
High school	9 (30)	21 (70)	
Diploma	4 (33.3)	8 (66.7)	
Bachelor	3 (9.4)	29 (90.6)	
Postgraduates	1 (11.1)	8 (88.9)	
**Subjects’ Occupation**			
Housewife	16 (31.4)	35 (68.6)	*0.09
Employee	2 (7.7)	24 (92.3)	
Teacher	1 (11.1)	8 (88.9)	
Others	1 (16.7)	5 (83.3)	
**Husbands’ Occupation**			
Employee	13 (21.7)	47 (78.3)	0.7
Enterpreneur	5 (29.4)	12 (70.6)	
Teacher	1 (20)	4 (80)	
Others	1 (10)	9 (90)	
**Address**			
Jakarta	10 (25)	30 (75)	0.53
BoDeTaBek	8 (22.9)	27 (77.1)	
Others	2 (11.8)	15 (88.2)	
**Mobile phone ownership**			
Yes	20 (21.7)	72 (78.3)	
No	0 (0)	0 (0)	
**Access to the internet**			
Yes	20 (21.7)	72 (78.3)	
No	0 (0)	0 (0)	
**Internet usage frequency**			
Rarely	6 (42.9)	8 (57.1)	*0.037
Min. 1 times a week	14 (17.9)	64 (82.1)	
**Exposure to family planning information**			
Never	11 (28.9)	27 (71.1)	0.137
Rarely	9 (20.5)	35 (79.5)	
Min. 1 times a week	0 (0)	10 (100)	

Data are presented by n (%).

## Discussion

This study was the first to investigate the unmet need for contraception in reproductive females with autoimmune diseases. Twenty (21.7%) subjects had an unmet need for contraception, while 72 (69.4%) were using contraception or planning for pregnancy. In the general population, the prevalence of unmet need contraception in Indonesia was 12% in 2019. ^9^ Meanwhile, Misnaniarti et al. found that the unmet need for contraception reached 59.09% in 2017. ^10^ Compared to other countries, the unmet need for contraception in India was 39%, in Africa was 22%, and in the Oceania region was 15%. ^3^

Women with autoimmune diseases usually do not use contraceptives. Contraception, especially hormonal contraception (pills, patches, injections, implants, or intrauterine progestin-releasing systems), can cause autoimmune diseases. In addition, autoimmune diseases can be affected by pregnancy. ^5^ Jungers et al. found that the use of hormonal contraception aggravates the symptoms in 44% of patients with SLE. ^11^ In addition, Julkunen et al. ^9^ found that 4 of 85 female SLE patients had major kidney involvement due to the use of combination oral contraception. In other autoimmune diseases, such as anti-phospholipid syndrome (APS), Julkunen et al. found two cases of vein thrombosis after the use of combination oral contraception. ^6^ In addition, Cervera et al. found that the prevalence of thrombosis was 30% in patients with APS who used hormonal contraception. ^12^

Women with autoimmune diseases are suggested to use natural or barrier contraception, such as abstinence, withdrawal method of contraception, or condoms. Apter et al. found that 22% of women with autoimmune disease used contraception inconsistently, while 53% used barrier contraception and 13% used an intrauterine device. ^13^ The use of contraception in women with autoimmune disease in those studies was similar to that in our study. Ahrendt et al. found that 54% of patients with SLE did not use any contraception. ^14^ Barrier contraception and intrauterine devices were relatively safe for female with autoimmune diseases. Sanchez-Guerrero et al. found that intrauterine devices did not affect the disease activity or flare incidence in patients with SLE. ^15^

In our study, most of the participants were Muslim (89.1%), bachelor’s degree (47.8%), housewives (55.4%), and lived in Jakarta (43.4%). All subjects had a handphone and access to the internet. Meanwhile, most husbands were bachelors (34.7%) and employees (65.2%). In general population, there were 2 main factors affecting unmet need contraception, including individual preference and disapproval for pregnancy prevention due to religion and culture. ^16, 17^ Other factors were sociodemographic factors, access to contraception, lack of information, and economic factors. ^18^ In women with autoimmune disease, the condition of their disease was affecting the unmet need contraception. ^19^

This study found no significant differences in religion between unmet need contraception and family planning. In Indonesia, there were 6 religions that recognized according to applicable law, including Islam, Christians, Catholics, Hinduism, Buddhism, and Kong Hu Cu. In our study, we included only subjects with Islam, Christians, and Buddha. Religious and cultural backgrounds can significantly affect how couples from different religious settings perceive and adopt contraceptive methods. Variations in the interpretations of religious doctrines concerning contraception can exist even within the same religion across different sects. ^20^

Islamic beliefs regarding contraception and family planning range from permissible to disapproval. However, only fundamentalist Muslims insist that any form of contraception is against God’s intention. ^21^ Protestants hold the view that marriages should be fruitful; however, once children are present, there are no restrictions on using contraception. Decisions regarding reproductive health, including the desired number of children, suitable conditions for using contraception, and the choice of contraceptive method, are made at the discretion of the couple. ^22^ Buddhism grants both men and women the freedom to employ any non-violent contraceptive methods. Family planning is acceptable and advocated as long as the intent behind using contraception is benevolent and causes no harm. ^23^

Our study found a significant difference in the wife’s education level between unmet need contraception and family planning in women with autoimmune diseases. However, we did not find a significant difference in the husbands’ education levels. Nuryana et al. found no significant difference in education between unmet need contraception and family planning. However, in the study by Nuryana et al., subjects with higher education were associated with a lower prevalence of unmet need contraception. ^24^ Education level influences people’s perception of some information, as well as how they search for information about something related to them. In addition, partner perception of contraception is also important in deciding which contraception method is used. ^25^

We did not find a significant difference in wife or husband occupation between unmet need contraception and family planning in women with autoimmune diseases. Most of the wives were housewives, whereas most of the husbands were employees. Nuryana et al. found that working status did not significantly associate with unmet needs for family planning. ^24^ Kholifatullah et al. also found that no significant association between employment status of unmet need women with intention to use contraception (p = 0.325). ^26^ Our result was similar to previous study. However, we grouped the participants based on their type of occupation in our study. We did not group the participants based on their working status. Most studies have grouped their subjects based on their working status.

We did not find a significant difference in residential areas between unmet need contraception and family planning. Saputri et al. also found no significant difference in residential area in unmet need of contraception. ^27^ Our study only differed in the area based on location and the location of our subjects living in urban cities, such as Jakarta, Bogor, Depok, Tangerang, and Bekasi, because we only conducted this study in a tertiary hospital located in the capital city of Indonesia. We did not conduct a multicenter study in other suburban or rural areas.

All subjects had handphones and access to the Internet because we conducted our study in a tertiary hospital located in the capital city of Indonesia. We found no significant differences in exposure to family planning on the Internet between unmet need contraception and family planning. However, we found that Internet usage frequency (OR 3.42; 95% CI 1.02-11.45; p = 0.037) was significantly associated with unmet need contraception in female with autoimmune diseases. Frequency of Internet usage can influence women’s fertility intentions and decisions regarding contraception. Liu et al. suggested that increased Internet use decreases fertility intentions partly because it provides women with greater access to information about family planning and contraception. Women gain more autonomy and information through increased internet access, which can lead to a reduced interest in traditional family roles and potentially lower fertility rates. ^28^

This study was the first to investigate the unmet need for contraception in reproductive females with autoimmune diseases. However, this study had several limitations. This study was conducted only in a tertiary hospital located in the capital city of Indonesia. We did not perform a multicenter study that included suburban and rural areas. Therefore, our participants had similar sociodemographic characteristics. In addition, our sample size was limited to the minimum required for the calculations. Therefore, further multicenter studies are required to confirm these findings.

This study found that subjects’ education level and Internet usage frequency were significantly associated with unmet need contraception in female with autoimmune disease. Other sociodemographic features did not significantly differ between unmet need contraception and family planning in women with autoimmune diseases; further, a larger and multicenter study is needed to confirm these findings.

## Ethical consideration

Participation was voluntary and uncompensated, and written informed consent was obtained. Ethical approval for the study was granted under the code KET-1387/UN2.F1/ETIK/PPM/00/02/2023 following review and authorization by the Research Committee Faculty of Medicine, Universitas Indonesia.

## Reporting guidelines

The reporting of this study adhered to the STROBE (Strengthening the Reporting of Observational Studies in Epidemiology) statement.

## Data Availability

Underlying dataZenodo: Raw data and demographic information of the study: Factors Affecting Unmet Need Contraception in Patients with Autoimmune Disease in Dr. Cipto Mangunkusumo Hospital, https://doi.org/10.5281/zenodo.16528112. ^29^ This project contains the following underlying data:
- Raw data and demographic information of the study Factors Affecting Unmet Need Contraception in Patients with Autoimmune Disease in Dr.xlsx. Zenodo: Raw data and demographic information of the study: Factors Affecting Unmet Need Contraception in Patients with Autoimmune Disease in Dr. Cipto Mangunkusumo Hospital, https://doi.org/10.5281/zenodo.16528112. ^29^ This project contains the following extended data:
- Informed Consent Form.docx - Questionnaire.docx - STROBE_Checklist.docx Data are available under the terms of the Creative Commons Attribution 4.0 International license (CC-BY 4.0).
