# Supplementary material for: Factors Affecting Unmet Need Contraception in Patients with Autoimmune Disease in Dr. Cipto Mangunkusumo Hospital
Source: F1000Res. 2025 Aug 22;14:813. [Version 1] doi: 10.12688/f1000research.167793.1 (PMC12749561; doi:10.12688/f1000research.167793.1)
Supplement: Peer review history

# Reviewer report: Reviewer response for version 1

**DOI:** 10.5256/f1000research.184936.r435486

**Published:** 2025-12-29

**Authors:** Hilma Putri Lubis

**Affiliations:** 1 Universitas Sumatera Utara, Medan, Indonesia

The study provides valuable insights into the influence of sociodemographic factors and Internet usage on fertility intentions. The study design is appropriate, utilizing a cross-sectional approach with clear inclusion criteria. The methodology is well-detailed, allowing for replication, and the statistical analyses are sound. The findings contribute valuable insights into how education and Internet access influence contraceptive needs.

 The manuscript offers key insights into the factors influencing unmet contraceptive needs among women with autoimmune diseases, highlighting the significant roles of education level and Internet usage frequency. It suggests that improved access to information and education can empower women to make informed decisions about contraception, potentially reducing unmet needs. Additionally, the study underscores the necessity for targeted reproductive health interventions that consider the unique challenges faced by this population. The study's limitations include a small sample size, drawn from a single tertiary hospital in an urban area, potentially limiting the generalizability of the findings. Additionally, the study did not include participants from suburban or rural areas, which may have different sociodemographic characteristics and unmet needs. Lastly, the age range starting at 15 years raises questions about the appropriateness of including younger participants in the context of unmet contraceptive needs.

Is the work clearly and accurately presented and does it cite the current literature?

Yes

If applicable, is the statistical analysis and its interpretation appropriate?

Yes

Are all the source data underlying the results available to ensure full reproducibility?

Yes

Is the study design appropriate and is the work technically sound?

Yes

Are the conclusions drawn adequately supported by the results?

Yes

Are sufficient details of methods and analysis provided to allow replication by others?

Yes

Reviewer Expertise:

Reproductive endocrinology, Infertility, Reproductive Genetics, Assisted Reproductive Technology (ART)

I confirm that I have read this submission and believe that I have an appropriate level of expertise to confirm that it is of an acceptable scientific standard.

# Reviewer report: Reviewer response for version 1

**DOI:** 10.5256/f1000research.184936.r419472

**Published:** 2025-11-13

**Authors:** Mulyanusa Amarullah Ritonga

**Affiliations:** 1 Padjadjaran University, Bandung, Indonesia

This study is unique because it encompasses a population rarely covered by reproductive health assessments. The inclusion criteria were somewhat surprising, as the participants were aged 15 to 49. The age of 15, in particular, remains unclear, whether this unmet need was due to the age of their partners or simply due to limited contraceptive information.

 Having more comprehensive data on the types of autoimmune diseases would have further strengthened the study's findings.

Is the work clearly and accurately presented and does it cite the current literature?

Yes

If applicable, is the statistical analysis and its interpretation appropriate?

Yes

Are all the source data underlying the results available to ensure full reproducibility?

Yes

Is the study design appropriate and is the work technically sound?

Yes

Are the conclusions drawn adequately supported by the results?

Yes

Are sufficient details of methods and analysis provided to allow replication by others?

Yes

Reviewer Expertise:

Reproductive Endocrinology and Infertility

I confirm that I have read this submission and believe that I have an appropriate level of expertise to confirm that it is of an acceptable scientific standard.
